# Vascular Endothelial Growth Factor Sequestration Enhances In Vivo Cartilage Formation

**DOI:** 10.3390/ijms18112478

**Published:** 2017-11-21

**Authors:** Carolina M. Medeiros Da Cunha, Valeria Perugini, Petra Bernegger, Matteo Centola, Andrea Barbero, Anna L. Guildford, Matteo Santin, Andrea Banfi, Ivan Martin, Anna Marsano

**Affiliations:** 1 Department of Surgery, University Hospital Basel, and Department of Biomedicine, University of Basel, Hebelstrasse 20, 4031 Basel, Switzerland; carol_medeiros@yahoo.com (C.M.M.D.C.); petrabernegger@gmail.com (P.B.); mcentola@anikatherapeutics.com (M.C.); andrea.barbero@usb.ch (A.B.); andrea.banfi@usb.ch (A.B.); Ivan.martin@usb.ch (I.M.); 2 Brighton Studies in Tissue-mimicry and Aided Regeneration, Centre for Regenerative Medicine and Devices, University of Brighton, Huxley Building Lewes Road, Brighton BN2 4GJ, UK; v.perugini@brighton.ac.uk (V.P.); anna@etaliauk.com (A.L.G.); M.Santin@brighton.ac.uk (M.S.)

**Keywords:** chondrogenesis, dendron, soluble VEGF receptor-2, nasal chondrocyte, collagen scaffold

## Abstract

Autologous chondrocyte transplantation for cartilage repair still has unsatisfactory clinical outcomes because of inter-donor variability and poor cartilage quality formation. Re-differentiation of monolayer-expanded human chondrocytes is not easy in the absence of potent morphogens. The Vascular Endothelial Growth Factor (VEGF) plays a master role in angiogenesis and in negatively regulating cartilage growth by stimulating vascular invasion and ossification. Therefore, we hypothesized that its sole microenvironmental blockade by either VEGF sequestration by soluble VEGF receptor-2 (Flk-1) or by antiangiogenic hyperbranched peptides could improve chondrogenesis of expanded human nasal chondrocytes (NC) freshly seeded on collagen scaffolds. Chondrogenesis of several NC donors was assessed either in vitro or ectopically in nude mice. VEGF blockade appeared not to affect NC in vitro differentiation, whereas it efficiently inhibited blood vessel ingrowth in vivo. After 8 weeks, in vivo glycosaminoglycan deposition was approximately two-fold higher when antiangiogenic approaches were used, as compared to the control group. Our data indicates that the inhibition of VEGF signaling, independently of the specific implementation mode, has profound effects on in vivo NC chondrogenesis, even in the absence of chondroinductive signals during prior culture or at the implantation site.

## 1. Introduction

The treatment of focal articular lesions still remains an unresolved clinical issue, despite the recent advancement of cell-based repair strategies [1]. Autologous articular chondrocyte-based implantation techniques have led to significant improvements in the clinical outcomes but cannot guarantee durable, functionally competent restoration of cartilage [2,3]. High inter-donor variability [4] and loss of phenotypic specificity during in vitro expansion [5,6] are critical issues which affect the chondrocyte potential to form stable cartilage in vivo.

Hyaline articular cartilage is avascular in nature and the maintenance of an avascular environment seems to be crucial for cartilage development and homeostasis [7]. The Vascular Endothelial Growth Factor (VEGF) is a crucial regulator of angiogenesis [8,9] and skeletal growth [10,11,12]: in particular, it mediates vascular invasion of hypertrophic cartilage during endochondral ossification. Thus, the blocking of angiogenesis by sequestering microenvironmental VEGF has been considered as a strategy to support and enhance cartilage repair, possibly in conjunction with cellular therapy. Kubo et al. [13] genetically modified muscle-derived stem cells to express soluble Flt-1, the extracellular cleaved form of VEGFR-1, which can act as decoy receptor resulting in angiogenesis blockade. These cells supported enhanced cartilage repair, but only when combined with a potent morphogen, namely BMP-4 [13]. Alternatively, the blocking of angiogenesis can be achieved by chondro-supportive scaffolds functionalized to release humanized VEGF antibodies [14] or with synthetic antiangiogenic peptides [15] able to form complexes with VEGF, thus preventing its binding to VEGFR-2 [16]. Recently, these linear VEGF blocker peptides with sequence WHLPFKC have been designed in a hyperbranched formulation able to expose the aptamer sequence at higher density to the surrounding biological microenvironment and protract the inhibitory effect on endothelial cell proliferation and sprouting in vitro [17,18,19]. However, the use of the resulting dendrons to support chondrogenesis has not yet been tested.

In this study, we aimed at investigating the effects of blocking angiogenesis on the cartilage-forming capacity of de-differentiated human nasal chondrocytes (NC). NC were chosen for their high clinical translation potential for articular cartilage repair, due to their safety [20,21], compatibility with articular cartilage repair [2], ease of harvest, and low donor site morbidity [8,22]. VEGF blockade was achieved by two different methods. The first one relied on NC transduction to overexpress the highest affinity soluble VEGF receptor, namely sFlk-1, and immediate loading on a collagen sponge [23], while the other was based on the direct functionalization of the collagen sponge with synthetic hyperbranched antiangiogenic peptides prior to NC loading. The anti-angiogenic and chondrogenic capacity of the resulting cellular constructs was assessed following implantation in a subcutaneous mouse model, which is highly vascularized and not supportive of chondrogenesis, unless implants have a strong intrinsic chondroinductive capacity [24].

## 2. Results

### 2.1. In Vitro Characterization of Engineered Nasal Chondrocytes

**Figure 1:**
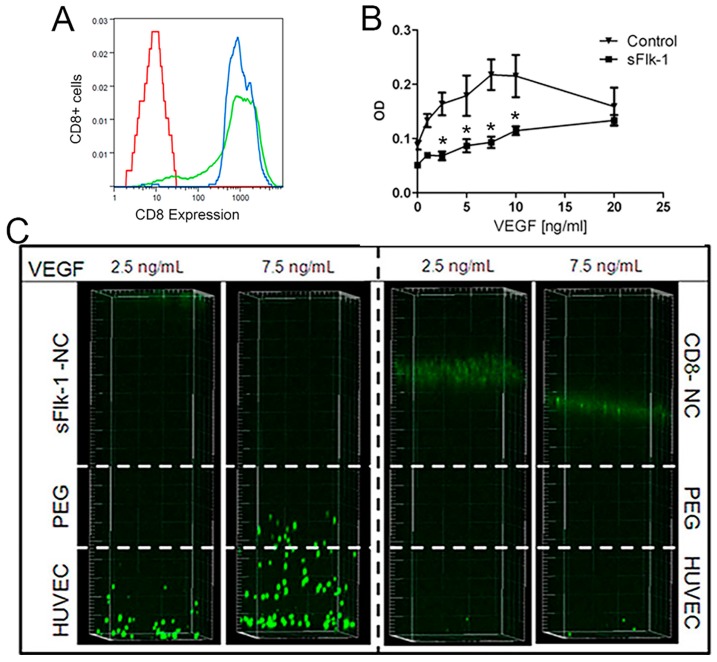
In vitro expression of CD8 and bio-activity of the sFlk-1 released by genetically modified human nasal chondrocytes. (**A**) Representative Fluorescence-activated cell sorting (FACS) plot shows that nasal chondrocytes (NC) had transduction efficiency above 80% (green-tinted plot) and populations of CD8-positive cells were purified after sorting (blue-tinted line). Not stained cells were acquired as control (in red). (**B**) Human umbilical vein endothelial cells (HUVEC) metabolic activity assay showing that sFlk-1-containing supernatants efficiently blocks Vascular Endothelial Growth Factor (VEGF). CD8-expressing NC supernatants were used as control (2 donors, *n* = 4). (**C**) HUVEC migration assay after 12 h showing that sFlk-1-expressing NC hampered HUVEC migration by blocking different gradients of VEGF, whereas CD8-only expressing NC allowed HUVEC migration. * *p* < 0.01.

Human nasal chondrocytes (NC) were transduced with a retroviral vector expressing a truncated version of murine CD8a, alone or linked to sFLk-1 in a bicistronic construct. CD8a-only transduced cell populations (control) and CD8a-sFlk-1-transduced cells were FACS-purified for positive CD8a expression to eliminate non-transduced cells (Figure 1A). The transduction efficiency for NC was 86.5 ± 9.2% (*n* = 3). The transduction process did not impair the proliferation potential of NC, as the total cell doublings achieved in 14 days by naïve, control, and sFlk-1 transduced NC were similar (9.5 ± 1.3, 8.8 ± 1.7, and 9.2 ± 2.0, respectively), consistent with previous findings [25]. ELISA quantification showed that monolayer-expanded transduced NC released 1.07 ± 0.41 ng/10^6^ cells/day of sFlk-1.

Human umbilical vein endothelial cells (HUVEC) proliferation and migration assays were performed in order to assess the activity of sFlk-1 released by transduced NC. Supernatants collected from monolayer expansion of sFlk-1-expressing NC significantly reduced HUVEC proliferation, assessed by quantification of the total metabolic activity, induced by concentrations of VEGF up to 10 ng/mL (Figure 1B).

sFlk-1-releasing NC also hindered the migration of HUVEC under two different concentrations of VEGF (namely 2.5 and 7.5 ng/mL). On the other hand, the control NC were permissive to the migration of HUVEC cells (Figure 1C). Taken together, these data show that sFlk-1 secreted by transduced NC is functionally active to block VEGF effects in vitro.

### 2.2. In Vitro Chondrogenesis

Naïve (Naïve), mock-sorted (Naïve_S), CD8a- (CD8, transduction control), and sFlk-1-CD8a-expressing (sFlk-1) cell populations were used to investigate the effect of the VEGF blockade and the transduction and sorting processes on the in vitro NC chondrocytic re-differentiation capacity (*n* = 3). Since hypoxic culture conditions were shown to influence in vitro cell chondrogenic capacity, the pellet culture was performed at either 2% or 20% of oxygen tension.

**Figure 2:**
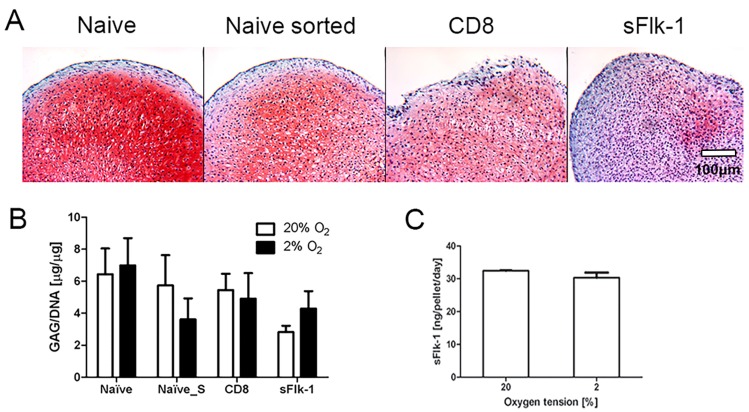
In vitro chondrogenesis of nasal chondrocytes. (**A**) Representative Safranin-O staining images of NC cultured in pellets at 20% of oxygen tension. Naïve, naïve mock-sorted (Naïve sorted), CD8 (control), and sFlk-1-releasing NC were cultured in vitro in pellets and analyzed for their chondrogenic potential. (**B**) Glycosaminoglycan (GAG) content of pellets generated by NC cultured at either 2% or 20% of oxygen tension (2 donors, *n* = 9). (**C**) Amount of mouse sFlk-1 released by sFlk-1-expressing NC-based pellets cultured at different oxygen tensions (2 donors, *n* = 4). No statistical significant difference has been found.

Cartilaginous extracellular matrix was formed in naïve, sorted naïve (naïve_S), and CD8 pellets, whereas lower deposition occurred in sFlk-1 pellets in 20% oxygen, as shown by the Safranin-O staining in 20% oxygen (Figure 2A). There was not a statistical significant difference between the glycosaminoglycan (GAG) amounts deposited by the naïve sorted and CD8-expressing NC, at either oxygen tension (Figure 2B). Nevertheless, a negative trend was present for the GAG/DNA ratio for Naïve-S at 2% of oxygen and for the sFlk-1 expressing NC at 20% of oxygen tension. Since the sorting and transduction process alone appeared not to affect the chondrogenic potential in vitro of NC, naïve cells were used as control group for the experiments in vivo. Mouse sFlk-1 was quantified by ELISA in the supernatant of NC after pellet culture. sFlk-1-transduced NC pellets expressed approximately 30 ng/pellet/day of sFlk-1 under 20% and 2% O_2_ (Figure 2C). Thus, sFlk-1 release was not affected by the different oxygen tensions used for pellet culture. As expected, murine sFlk-1 expression in naïve and CD8- transduced cells remained undetected.

### 2.3. In Vivo Blocking of Angiogenesis

**Figure 3:**
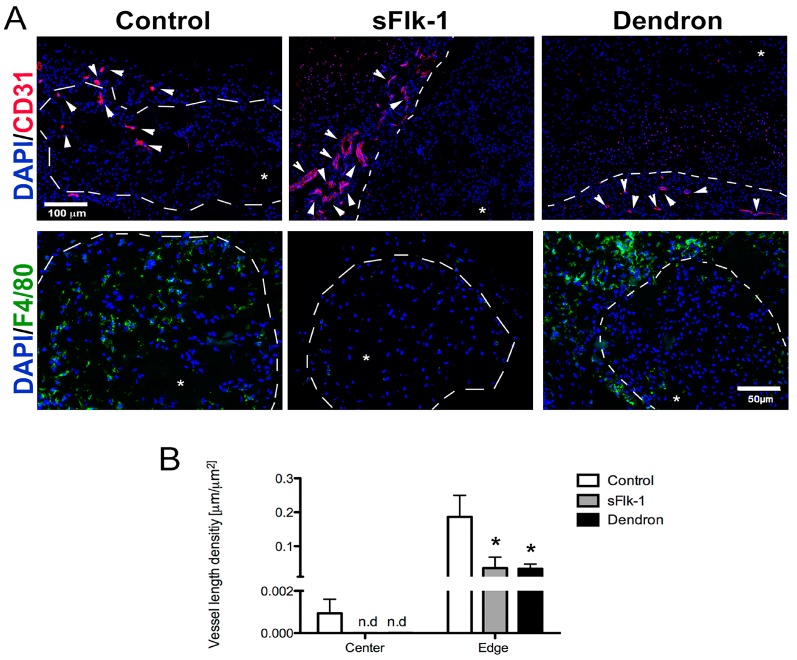
In vivo blocking of angiogenesis. (**A**) Representative immunofluorescence pictures of control (naïve NC), sFlk-1, and dendron-based constructs stained either in red for PECAM (CD31), an endothelial marker, (first raw, scale bar = 100 µm) or in green for F4/80, a macrophage marker (second raw, scale bar = 50 µm). Cell nuclei were stained in blue with DAPI (**A**). White arrows indicate the vascular structures, whereas white dotted lines mark the boundary between the implant and the surrounding host tissue. Asterisks indicate the implant area. (**B**) Quantification of the vessel length density in µm/µm^2^ at the center or edge of the implant area of naïve, sFlk-1, and dendron-based constructs (1 donor, *n* = 4). * *p* < 0.01.

We first investigated whether sFlk-1 and the dendronized peptide of WHLPFKC were capable of blocking VEGF in vivo. Vessel ingrowth within the implant was investigated 8 weeks after ectopic subcutaneous implantation of cell-loaded constructs in nude mice. In both sFlk-1-expressing and dendron-based constructs, CD31-positive blood vessels could be observed only in the host tissue surrounding the constructs, proving their ability to efficiently block angiogenesis within the implant area (Figure 3A). Only in the control (naïve NC) samples could CD31-positive vessels be observed both at the edge and within the center of the implants, indicating vascular invasion (Figure 3A,B). Mouse-origin macrophages, positive for F4/80, were highly present within the center and periphery of control samples, whereas in the VEGF sequestrating groups, their infiltration was very low and limited to the edges of the implants (Figure 3A). At the edge of the constructs treated with dendrons or loaded with sFlk-1 expressing NC, we noticed a reduced number of vessel density as compared to the control, showing the potential to block host vasculature ingrowth also at the periphery of the implant. Taken together, these data show that both sFlk-1 and the dendronized WHLPFKC peptide were effective in blocking in vivo VEGF activity on both vessel invasion and macrophage recruitment.

### 2.4. In Vivo Chondrogenesis

Implanted tissues were also assessed for their in vivo chondrogenic potential. In vivo chondrogenesis was assessed after direct implantation of freshly seeded NC on Ultrafoam^®^ scaffolds. Since the cell transduction and sorting did not influence the chondrogenic capacity of nasal chondrocytes, we used only naïve cells as controls. Constructs engineered by CD8-expressing NC indeed displayed a strong positive staining for GAG and type II collagen, and resulted to be almost negative for the type X collagen also following 8 weeks in vivo (Supplementary Materials Figure S1).

**Figure 4:**
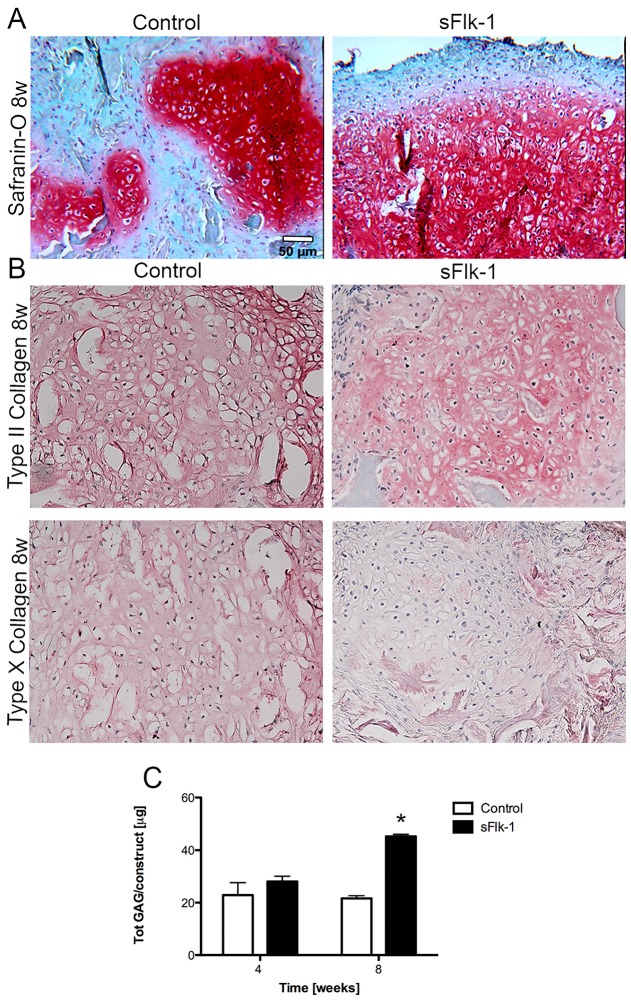
In vivo chondrogenesis of nasal chondrocytes—cell-based gene therapy approach. (**A**) Safranin-O staining for naïve (control) and sFlk-1-releasing NC after 8 weeks in vivo. (**B**) Types II and X collagen stainings for naïve and sFlk-1 NC at 8 weeks in vivo. (**C**) Total amount of GAG deposited in vivo at 4 and 8 weeks by naïve and sFlk-1 expressing NC (2 donors, *n* = 8). * *p* < 0.01. Scale bars = 50 µm.

Cartilaginous extracellular matrix, detected by positive staining for Safranin-O, was formed by both control (naïve) and sFlk-1-expressing cells already after 4 weeks in vivo. Constructs generated with sFlk-1 cells contained larger Safranin-O areas (Figure 4A) as comparted to those generated by controls cells and accumulated larger amounts of GAG (Figure 4C)*.* After 8 weeks in vivo, similarly to control samples, sFlk-1 cartilage-like constructs were positive for type II (hyaline cartilage-specific marker) and negative for type X (hypertrophic marker) collagens in the center of the implant where cartilage was formed, indicating a stable phenotype (Figure 4B).

**Figure 5:**
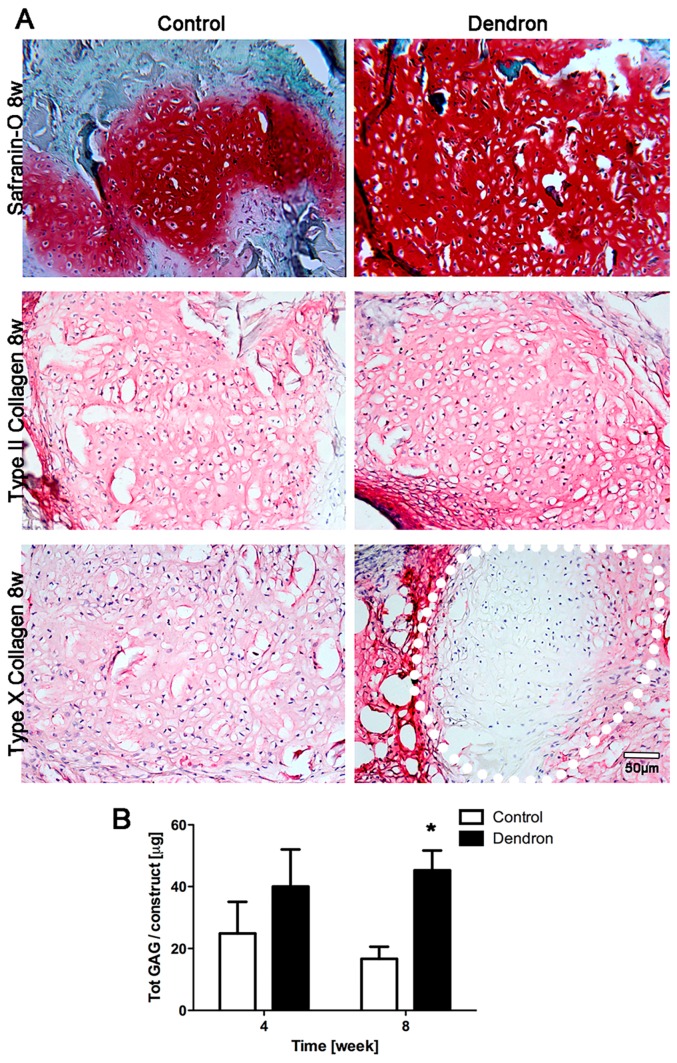
In vivo chondrogenesis of nasal chondrocytes-dendron-functionalized approach. (**A**) Safranin-O, types II and X collagen staining for control (naïve NC) and dendron-based constructs at 8 weeks in vivo. Dotted white line indicates the cartilaginous part of the dendron based implants. Scale bar = 50 µm. (**B**) Total GAG production in vivo at 4 and 8 weeks by control (naïve NC) and dendron-based constructs (1 donor, *n* = 5). * *p* < 0.01.

In vivo chondrogenesis of NC was also investigated with dendron-based constructs. The enhanced cartilage forming capacity of the dendron-based construct was evident after 8 weeks in vivo. Safranin-O staining was intense for both control and dendron-based constructs, but the latter displayed a more uniform spatial distribution of GAG (Figure 5A). Biochemical analyses of GAG deposition confirmed the finding that the anti-angiogenic dendronized peptide significantly enhanced the in vivo cartilage forming capacity of NC at 8 weeks (Figure 5B). Type II collagen was immunodetected in both groups. In the dendron-based constructs, the central part was mainly cartilaginous and expressed low type X collagen. At the edges in contact with the host connective tissue instead type X was highly expressed (Figure 5A).

## 3. Discussion

In this study, we demonstrated that two unrelated methods to block angiogenesis have a great impact on the in vivo chondrogenic potential of non-pre-committed NC by enhancing glycosaminoglycan (GAG) deposition as compared to controls. VEGF blockade in the surroundings of the implanted human NC, in order to inhibit blood vessel ingrowth within the implant area, might also reduce the risk of resorption of non-mature constructs [14] and supports chondrogenesis. Anti-angiogenic approaches showed improvement in cartilage repair by either applying an adeno-associated virus carrying the chondromodulin-1 gene [26] or by injecting mouse muscle-derived stem cells genetically modified to express the soluble version of Flt-1 combined with morphogenic factors [13] in knee cartilage lesions. These previous studies differ from the present investigation because of both the in vivo animal model adopted and the target cell types used: animal-origin native articular versus human de-differentiated nasal chondrocytes. While Kubo’s study indeed aimed at investigating the effects on cartilage repair of several growth factors, released by the genetically modified stem cells (which served mainly as a delivery system), Ref.[13] the goal of the present study was to generate a protected and chondro-permissive environment to allow sFlk-1-expressing NC re-differentiation. However, as in the previously mentioned studies, the enhanced chondrogenesis observed by adopting antiangiogenic strategies appears to be associated only with paracrine signaling to the host environment, since the in vitro chondrogenic potential was not directly affected by VEGF blockade or other antiangiogenic factors, such as endostatin [27]. Since the effects of VEGF blockade on chondrogenesis take place when host vascular invasion is prevented from happening in vivo [23], differences in NC differentiation potential were not observed in vitro. Paracrine VEGF signaling is crucial for angiogenesis to take place, including effects on EC proliferation, survival, migration, and endothelial differentiation [28]. Since one of the immediate consequences of inhibition of angiogenesis is the generation of a hypoxic environment, which is a key regulator of chondrogenesis during limb development [29], this study also investigated the in vitro effect of VEGF blockade at low oxygen tension. Based on the in vitro results of this investigation, it can be concluded that the mechanisms of chondrogenesis in vivo are controlled by factors beyond the combined effect of hypoxia and morphogen gradient availability. The lack of nutrients is also a direct consequence of the absence of blood vessel supply; low glucose culture has been shown to regulate the oxygen tension within engineered tissues [30] and the chondrogenesis capacity of chondrocytes [31]. Hence, glucose availability may be directly involved in NC chondrogenesis. The lack of vessels could prevent the infiltration of immune cells, such as macrophages, which can induce host reaction against the implant and bring about its premature resorption. Luo et al., have demonstrated that a milder inflammatory response by the host tissue occurs when a 2-week in vitro pre-cultured chondrocyte-based construct is implanted in vivo compared to a “no pre-culture group” [32]. Other studies have also reported that in vitro preconditioning can mitigate the post-implantation inflammatory reaction and how cartilage maturation is correlated with a different inflammatory chemokine production [33]. The present findings clearly highlight the different levels of macrophage invasion within the control groups, whereas the antiangiogenic groups (sFlk-1 and dendron-associated implants) displayed a diminished macrophage invasion, improving the cartilage quality and hampering its resorption through inflammatory reactions [14].

Thus, vessel ingrowth and the subsequent inflammatory reaction that take place in immature cartilaginous engineering constructs appear highly detrimental for cartilage formation. A clear control over host blood vessel invasion and inflammatory responses is likely to avoid tissue resorption at a later stage of the treatment [14]. The here presented results suggest that the blockage of vessel ingrowth also decreases the infiltration of inflammatory cells allowing the formation of stable cartilage up to 8 weeks in vivo without the need of a pre-culture period, being therefore highly attractive for clinical applications. However, in view of translating this concept into clinical approaches, further investigations are needed. In the present study, the effects of sFlk-1 and the WHLPFKC peptide on cartilage formation by NC were investigated in an ectopic environment, which is highly vascularized and permissive but not inductive to chondrogenesis [24], thus providing a reliable proof of principle of the clinical potential of these antiangiogenic treatments. However, further investigations in an orthotopic environment, where various physiological stimuli (e.g., mechanical loading and exposure of the NC to the cartilaginous milieu) are taken into account, are necessary to prove the cartilage long-term stability. Although both approaches here investigated efficiently blocked angiogenesis and supported chondrogenesis up to 8 weeks in vivo, they differ in the time of efficacy and in their clinical translational potential. Cell-based gene therapy indeed guarantees a sustained release of proteins, whereas the scaffold functionalized with the antiangiogenic peptide might be limited to the dendron reservoir capacity offered by the implanted scaffold. The time during which the antiangiogenic effect needs to be maintained in order to induce chondrogenesis still needs to be elucidated. However, the above proposed study in an orthotopic model will be highly relevant to fully assess the clinical potential of each approach. If a sustained antiangiogenic effect was found to be essential, retroviral vectors might be substituted by lentiviruses, which have become the vector of choice in clinical applications requiring indefinite expression of the therapeutic gene due to their significantly better safety profile [34].

## 4. Materials and Methods

### 4.1. Cell Isolation and Transduction

Human nasal septal cartilage biopsies were harvested from two donors during plastic surgical procedures in accordance to the local ethical committee and following informed consent. The nasal cartilage was digested with a 0.15% type II collagenase solution (1 mL/100 mg of tissue, Worthington Biochemical Corporation, Lakewood, NJ, USA) for 22 h at 37 °C. Nasal chondrocytes (NC) were cultured at 37 °C and 5% CO_2_ at a density of 5000 cells/cm^2^ in high glucose Dulbecco’s Modified Eagle Medium (DMEM) supplemented with 10% FBS, 1% Hepes (Gibco), 1% penicillin-streptomycin solution (Gibco, Zug, Switzerland), 1% sodium pyruvate (Gibco), 0.29 mg/mL L-glutamate (complete medium, CM) and with 5 ng/mL fibroblast growth factor-2 (FGF-2), and 1 ng/mL of transforming growth factor β1 (TGFβ1) (R&D Systems, Abingdon, UK) [2].

The NC were transduced with retroviral vectors carrying the gene for mouse sFlk-1 linked through an internal ribosomal entry site (IRES) to a non-functional truncated version of mouse CD8a, as a convenient syngenic cell surface marker to allow Fluorescence-Activated Cell Sorting (FACS) [35]. For control purposes, NC transduced only with the truncated murine CD8a and either mock-sorted or naïve cells were used. Cells were transduced at high efficiency according to a published protocol [25,36]. Briefly, the NC were incubated with retroviral vector supernatants supplemented with 8 µg/mL polybrene (Sigma-Aldrich, Buchs, Switzerland) for 5 min at 37 °C and then centrifuged at 1100× *g* for 30 min at room temperature (RT), followed by fresh medium replacement. Cells were then expanded for two additional passages.

### 4.2. In Vitro 3D Pellet Culture

3D NC pellets were formed as previously described [37] and cultured in a serum-free medium (SFM), which consists of DMEM, supplemented with 1% Hepes, 1% penicillin–streptomycin solution, 1% sodium pyruvate, 1% Insulin–transferrin–sodium selenite (ITS + 1, Sigma-Aldrich), 1% human serum albumin (HSA 100×, Invitrogen, Basel, Switzerland), 0.1 mM ascorbic acid 2-phosphate (Invitrogen), and dexamethasone 10^−7^ M (Invitrogen), then supplemented with 10 ng/mL of TGFβ-1. The pellets (made of not transduced (naïve), mock-sorted (naïve sorted), or cells transduced for expressing truncated CD8 alone (CD8) or in combination with sFlk-1) were cultured for 2 weeks at 2% or 20% oxygen tension. The supernatants of the pellet culture were collected and used for human sFlk-1 quantification (described below).

### 4.3. Synthesis and Characterization of Peptide VEGF Blockers

FFG3K (WHLPFKC)_16_ was assembled on Tenta Gel S-NH_2_ resin using an acid labile Rink amide linker (Iris Biotech GmbH, Marktredwitz, Germany) by a modified microwave based solid phase peptide synthesiser (Biotage, Ystrad Mynach, Hengoed, UK) and then isolated using 94% *v*/*v* TFA (Fisher Scientific, Loughborough, UK), 2.5% *v*/*v* deionised H_2_O, 1% *v*/*v* 1,2-ethandithiol (Sigma Aldrich Co., Ltd., Irvine, Ayrshire, UK), and 2.5% *v*/*v* trisopropyl silane (TIPS) (Sigma Aldrich Co., Ltd., Irvine, Ayrshire, UK). The dendron was characterized by analytical HPLC (Waters TM 717 plus Autosampler, Elstree, Herts, UK), mass spectrometry (MS) (Bruker microTOF, Coventry, UK), and gravimetric analysis as previously reported [17].

### 4.4. 3D Construct Preparation 

A commercially available type I collagen-based sponge (Ultrafoam^TM^, Bard Medica S.A., Zurich, Switzerland) of 3 mm thickness was punched into 6 mm diameter disks and then seeded at the density of 1.7 × 10^6^ cells per scaffold. For the cell-based gene approach, NC transduced to express sFlk-1 linked to CD8a were used. Cells expressing CD8a alone and naïve cells served as control.

Alternatively, for the dendron-functionalized approach, NC were loaded in the presence of WHLPFKC at a concentration of 1.62 nM, previously reported to produce an anti-angiogenic effect in vitro [17,18]. All the constructs were incubated with CM before for 24 h prior implantation. Naïve NC-based constructs without the addition of WHLPFKC were used as control group.

### 4.5. In Vivo Ectopic Implantation

Cell-based constructs were implanted ectopically (in subcutaneous pockets) in nude mice (CD-1 nude/nude, Charles River). The mice were sacrificed with CO_2_ at 4 and 8 weeks after the implantation. Animals were treated in agreement with Swiss legislation and according to a protocol approved by the Veterinary Office of Canton Basel-Stadt (permission number: 1797, 1 January 2016). All constructs were implanted in vivo 24 h after the cell seeding.

### 4.6. Flow Cytometry

The percentage of population positive for CD8a in the sFlk-1-releasing NC and control NC was assessed by staining with a fluorescein isothiocyanate-conjugated anti-mouse CD8a antibody (clone 53-6.7; BD Biosciences, Allschwil, Switzerland) using 0.8 μg of antibody/10^6^ cells in 200 μL of PBS with 5% bovine serum albumin. [35] Data were acquired with a FACSCalibur flow cytometer (BD Biosciences, Allschwil, Switzerland) and analyzed using FlowJo software (Tree Star, software version 10, Ashland, OR, USA).

### 4.7. Quantification of Murine sFlk-1

Supernatants were collected from monolayer expanded NC or pellets after 4 h or two days of culture, respectively, and were used for murine sFlk-1 quantification using an ELISA (enzyme-linked immunosorbent assay) Quantikine immunoassay kit (R&D Systems, Abingdon, UK). Data were presented as quantity in ng per pellet normalized to the time of incubation.

### 4.8. Biochemistry

Engineered constructs were digested with protease K for 15 h at 56 °C [38]. The glycosaminoglycan (GAG) and DNA contents were measured spectrophotometrically using dimethylmethylene blue [39] and the CyQUANT Kit (Molecular Probes, Eugene, OR, USA), respectively. GAG contents were normalized to the amount of DNA for the in vitro experiments. In the in vivo studies GAG amount was instead presented as total amount per construct.

### 4.9. HUVEC Proliferation Assay 

Human umbilical vein endothelial cells (HUVEC) were cultured in 96-well plates overnight in growth medium (GM), consisting of M199 supplemented with 20% fetal bovine serum (FBS), 100 mg/mL endothelial cells growth supplement, 50 IU/mL heparin, 100 IU/mL penicillin, and 100 mg/mL streptomycin, at a density of 5000 cells/well. The next day, GM was replaced with assay medium (AM), consisting of M199 with 5% FBS, 10 IU/mL heparin, and 1% penicillin/streptomycin, supplemented with recombinant human VEGF (R&D Systems) at different concentrations between 0 and 50 ng/mL, and mixed with the supernatant derived from either control or sFlk-1 expressing NC. [14] HUVEC proliferation was then assessed by using MTS [3-(4,5-dimethylthiazol-2-yl)-5-(3-carboxymethoxyphenyl)-2-(4-sulfophenyl)-2H-tetrazolium] (Promega, Dübendorf, Switzerland). The HUVEC were incubated with MTS for 2 h at 37 °C and the measurement of the absorbance of the formazan was carried out at 490 nm.

### 4.10. HUVEC Migration Assay

HUVEC encapsulation, along with either sFlk-1-expressing or CD8-only expressing NC, was performed by using a synthetic matrix metalloproteinase (MMP)-degradable extracellular matrix (ECM) functionalized with RGD peptides to promote degradation and cell adhesion and migration, respectively (kindly provided by QGel S.A., Lausanne, Switzerland). The HUVEC were first labelled with syto^®^13 in green and then seeded at a density of 13 × 10^4^ cells/well on collagen-coated culture plates. After HUVEC adhesion and proliferation overnight in GM, 100 µL of QGel^®^ with RGD was polymerized, followed by 100 µL of QGel^®^ without RGD, and 100 µL of sFlk-1-expressing or naïve NC embedded in QGel^®^ with RGD. The next day, the growth medium was replaced by AM supplemented either by 0, 2.5, or 7.5 ng/mL of human recombinant VEGF. HUVEC migration was monitored with time laps for 12 h, using Zeiss LSM 710 confocal microscope. Representative images of the culture wells following 12 h were taken to show whether the HUVEC migrated.

### 4.11. Histology

Explanted tissues were fixed with 4% formaldehyde and embedded in paraffin. Sections (5 µm thick) were stained for Safranin-O and for immunohistochemistry for types II and X collagen. The primary antibodies used were mouse anti-collagen II (MP Biomedicals, clone II-4C11), and mouse anti-collagen X (Abcam, Cambridge, UK). As a secondary antibody, goat anti-mouse IgG/Biotin (Dako, Basel, Switzerland) was used.

Explants dedicated to immunofluorescence analysis were fixed with 1% paraformaldehyde (Sigma-Aldrich, Buchs, Switzerland) in PBS, then incubated in 10% *w*/*v* sucrose (Sigma-Aldrich, Buchs, Switzerland) and, finally, embedded in OCT compound (Sakura Finetek) frozen in freezing isopentane (Sigma-Aldrich, Buchs, Switzerland). Immunofluorescence staining was performed on the 10 μm-thick sections with the following primary antibodies and dilutions: rat anti-mouse F4/80 antibody (Caltag, RM2900, 1:200) or rat anti-mouse PECAM-1 (CD31, BD 553370, 1:100). Fluorochrome-conjugated secondary antibodies were used at 1:200 (Invitrogen, Basel, Switzerland).

For immunohistochemistry staining, the intensity and the nonspecific binding of the secondary antibody were checked by performing negative controls, consisting in the staining without the use of the primary antibodies.

### 4.12. Vessel Quantification

The amount of vessels was quantified tracing CD31-positive vessels by using CellSens software (Olympus). Briefly, three representative fluorescence images were acquired from the center and the periphery of each construct (*n* = 3 samples/group) (BX63 microscope, Olympus). The vessel length density (VLD) was determined by dividing the total traced length of vessels identified in the fields by the area of the analysed images.

### 4.13. Statistical Analysis

The data were analysed with the statistical software Prism 5.0 (GraphPad Software Inc., La Jolla, CA, USA) using one-way analysis of variance (ANOVA) with Bonferroni correction (*p* < 0.05). Data are presented as mean ± standard deviation (SD).

## Supplementary Materials

Supplementary materials can be found at www.mdpi.com/1422-0067/18/11/2478/s1.

## References

[1] Dunkin B.S., Lattermann C. (2013). New and Emerging Techniques in Cartilage Repair: MACI. Oper. Tech. Sports Med..

[2] Pelttari K., Pippenger B., Mumme M., Feliciano S., Scotti C., Mainil-Varlet P., Procino A., von Rechenberg B., Schwamborn T., Jakob M. (2014). Adult human neural crest-derived cells for articular cartilage repair. Sci. Transl. Med..

[3] Hunziker E.B. (2009). The elusive path to cartilage regeneration. Adv. Mater..

[4] Wolf F., Haug M., Farhadi J., Candrian C., Martin I., Barbero A. (2008). A low percentage of autologous serum can replace bovine serum to engineer human nasal cartilage. Eur. Cells Mater..

[5] Dell’Accio F., De Bari C., Luyten F.P. (2001). Molecular markers predictive of the capacity of expanded human articular chondrocytes to form stable cartilage in vivo. Arthritis Rheum..

[6] Schnabel M., Marlovits S., Eckhoff G., Fichtel I., Gotzen L., Vécsei V., Schlegel J. (2002). Dedifferentiation-associated changes in morphology and gene expression in primary human articular chondrocytes in cell culture. Osteoarthr. Cartil..

[7] Descalzi Cancedda F., Melchiori A., Benelli R., Gentili C., Masiello L., Campanile G., Cancedda R., Albini A. (1995). Production of angiogenesis inhibitors and stimulators is modulated by cultured growth plate chondrocytes during in vitro differentiation: Dependence on extracellular matrix assembly. Eur. J. Cell Biol..

[8] Ferrara N., Gerber H.P. (2001). The role of vascular endothelial growth factor in angiogenesis. Acta Haematol..

[9] Carmeliet P., Collen D. (2000). Molecular basis of angiogenesis. Role of VEGF and VE-cadherin. Ann. N. Y. Acad. Sci..

[10] Zelzer E., McLean W., Ng Y.S., Fukai N., Reginato A.M., Lovejoy S., D’Amore P.A., Olsen B.R. (2002). Skeletal defects in VEGF(120/120) mice reveal multiple roles for VEGF in skeletogenesis. Development.

[11] Gerber H.P., Vu T.H., Ryan A.M., Kowalski J., Werb Z., Ferrara N. (1999). VEGF couples hypertrophic cartilage remodeling, ossification and angiogenesis during endochondral bone formation. Nat. Med..

[12] Street J., Bao M., deGuzman L., Bunting S., Peale F.V., Ferrara N., Steinmetz H., Hoeffel J., Cleland J.L., Daugherty A. (2002). Vascular endothelial growth factor stimulates bone repair by promoting angiogenesis and bone turnover. Proc. Natl. Acad. Sci. USA.

[13] Kubo S., Cooper G.M., Matsumoto T., Phillippi J.A., Corsi K.A., Usas A., Li G., Fu F.H., Huard J. (2009). Blocking vascular endothelial growth factor with soluble Flt-1 improves the chondrogenic potential of mouse skeletal muscle-derived stem cells. Arthritis Rheum..

[14] Centola M., Abbruzzese F., Scotti C., Barbero A., Vadalà G., Denaro V., Martin I., Trombetta M., Rainer A., Marsano A. (2013). Scaffold-based delivery of a clinically-relevant anti-angiogenic drug promotes the formation of in vivo stable cartilage. Tissue Eng. Part A.

[15] Foy K.C., Liu Z., Phillips G., Miller M., Kaumaya P.T. (2011). Combination treatment with HER-2 and VEGF peptide mimics induces potent anti-tumor and anti-angiogenic responses in vitro and in vivo. J. Biol. Chem..

[16] Erdag B., Balcioglu K.B., Kumbasar A., Celikbicak O., Zeder-Lutz G., Altschuh D., Salih B., Baysal K. (2007). Novel short peptides isolated from phage display library inhibit vascular endothelial growth factor activity. Mol. Biotechnol..

[17] Meikle S.T., Perugini V., Guildford A.L., Santin M. (2011). Synthesis, characterisation and in vitro anti-angiogenic potential of dendron VEGF blockers. Macromol. Biosci..

[18] Perugini V., Guildford A.L., Silva-Correia J., Oliveira J.M., Meikle S.T., Reis R.L., Santin M. (2016). Anti-angiogenic potential of VEGF blocker dendron loaded on to gellan gum hydrogels for tissue engineering applications. J. Tissue Eng. Regen. Med..

[19] Reitmaier S., Kreja L., Gruchenberg K., Kanter B., Silva-Correia J., Oliveira J.M., Reis R.L., Perugini V., Santin M., Ignatius A. (2014). In vivo biofunctional evaluation of hydrogels for disc regeneration. Eur. Spine J..

[20] Fulco I., Miot S., Haug M.D., Barbero A., Wixmerten A., Feliciano S., Wolf F., Jundt G., Marsano A., Farhadi J. (2014). Engineered autologous cartilage tissue for nasal reconstruction after tumour resection: An observational first-in-human trial. Lancet.

[21] Mumme M., Barbero A., Miot S., Wixmerten A., Feliciano S., Wolf F., Asnaghi A.M., Baumhoer D., Bieri O., Kretzschmar M. (2016). Nasal chondrocyte-based engineered autologous cartilage tissue for repair of articular cartilage defects: An observational first-in-human trial. Lancet.

[22] Siegel N.S., Gliklich R.E., Taghizadeh F., Chang Y. (2000). Outcomes of septoplasty. Otolaryngol. Head Neck Surg..

[23] Marsano A., Medeiros da Cunha C.M., Ghanaati S., Gueven S., Centola M., Tsaryk R., Barbeck M., Stuedle C., Barbero A., Helmrich U. (2016). Spontaneous In Vivo Chondrogenesis of Bone Marrow-Derived Mesenchymal Progenitor Cells by Blocking Vascular Endothelial Growth Factor Signaling. Stem Cells Transl. Med..

[24] Scott M.A., Levi B., Askarinam A., Nguyen A., Rackohn T., Ting K., Soo C., James A.W. (2012). Brief review of models of ectopic bone formation. Stem Cells Dev..

[25] Helmrich U., Marsano A., Melly L., Wolff T., Christ L., Heberer M., Scherberich A., Martin I., Banfi A. (2012). Generation of human adult mesenchymal stromal/stem cells expressing defined xenogenic vascular endothelial growth factor levels by optimized transduction and flow cytometry purification. Tissue Eng. Part C Methods.

[26] Klinger P., Surmann-Schmitt C., Brem M., Swoboda B., Distler J.H., Carl H.D., von der Mark K., Hennig F.F., Gelse K. (2011). Chondromodulin 1 stabilizes the chondrocyte phenotype and inhibits endochondral ossification of porcine cartilage repair tissue. Arthritis Rheum..

[27] Jeng L., Olsen B.R., Spector M. (2010). Engineering endostatin-producing cartilaginous constructs for cartilage repair using nonviral transfection of chondrocyte-seeded and mesenchymal-stem-cell-seeded collagen scaffolds. Tissue Eng. Part A.

[28] Lee S., Chen T.T., Barber C.L., Jordan M.C., Murdock J., Desai S., Ferrara N., Nagy A., Roos K.P., Iruela-Arispe M.L. (2007). Autocrine VEGF signaling is required for vascular homeostasis. Cell.

[29] Araldi E., Schipani E. (2010). Hypoxia, HIFs and bone development. Bone.

[30] Heywood H.K., Bader D.L., Lee D.A. (2006). Glucose concentration and medium volume influence cell viability and glycosaminoglycan synthesis in chondrocyte-seeded alginate constructs. Tissue Eng..

[31] Heywood H.K., Nalesso G., Lee D.A., Dell’accio F. (2014). Culture expansion in low-glucose conditions preserves chondrocyte differentiation and enhances their subsequent capacity to form cartilage tissue in three-dimensional culture. Biores. Open Access.

[32] Luo X., Zhou G., Liu W., Zhang W.J., Cen L., Cui L., Cao Y. (2009). In vitro precultivation alleviates post-implantation inflammation and enhances development of tissue-engineered tubular cartilage. Biomed. Mater..

[33] Francioli S., Cavallo C., Grigolo B., Martin I., Barbero A. (2011). Engineered cartilage maturation regulates cytokine production and interleukin-1β response. Clin. Orthop. Relat. Res..

[34] Montini E., Cesana D., Schmidt M., Sanvito F., Bartholomae C.C., Ranzani M., Benedicenti F., Sergi L.S., Ambrosi A., Ponzoni M. (2009). The genotoxic potential of retroviral vectors is strongly modulated by vector design and integration site selection in a mouse model of HSC gene therapy. J. Clin. Investig..

[35] Misteli H., Wolff T., Füglistaler P., Gianni-Barrera R., Gürke L., Heberer M., Banfi A. (2010). High-throughput flow cytometry purification of transduced progenitors expressing defined levels of vascular endothelial growth factor induces controlled angiogenesis in vivo. Stem Cells.

[36] Banfi A., Springer M.L., Blau H.M. (2002). Myoblast-mediated gene transfer for therapeutic angiogenesis. Methods Enzymol..

[37] Barbero A., Ploegert S., Heberer M., Martin I. (2003). Plasticity of clonal populations of dedifferentiated adult human articular chondrocytes. Arthritis Rheum..

[38] Hollander A.P., Heathfield T.F., Webber C., Iwata Y., Bourne R., Rorabeck C., Poole A.R. (1994). Increased damage to type II collagen in osteoarthritic articular cartilage detected by a new immunoassay. J. Clin. Investig..

[39] Farndale R.W., Buttle D.J., Barrett A.J. (1986). Improved quantitation and discrimination of sulphated glycosaminoglycans by use of dimethylmethylene blue. Biochim. Biophys. Acta.

