# Supplementary figures and images for: Vascular Endothelial Growth Factor Sequestration Enhances In Vivo Cartilage Formation
Source: Int J Mol Sci. 2017 Nov 21;18(11):2478. doi: 10.3390/ijms18112478 (PMC5713444; doi:10.3390/ijms18112478)

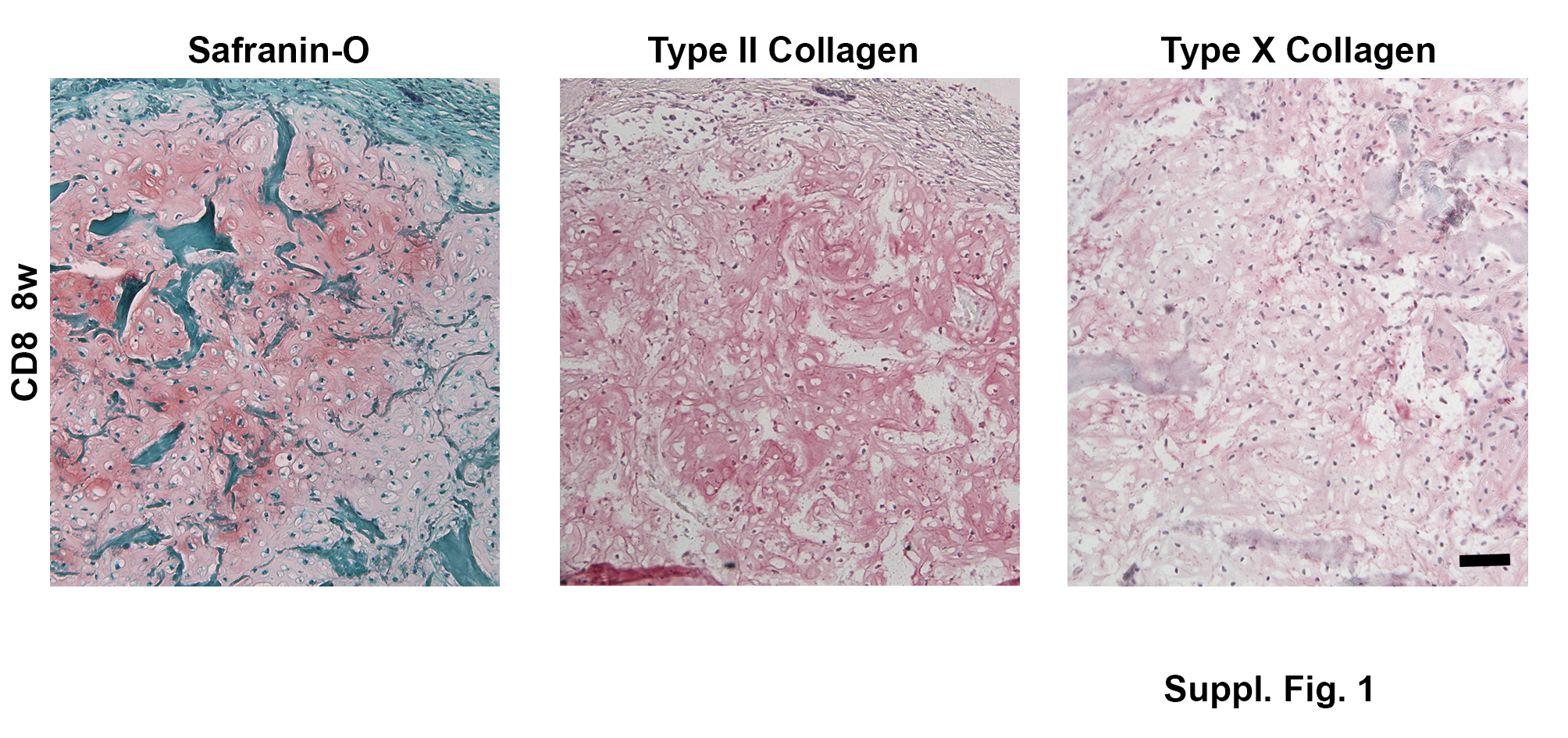

Supplement: Supplementary file 1 [file ijms-18-02478-s001.tif]
